# BMP3 Alone and Together with TGF-β Promote the Differentiation of Human Mesenchymal Stem Cells into a Nucleus Pulposus-Like Phenotype

**DOI:** 10.3390/ijms160920344

**Published:** 2015-08-27

**Authors:** Xiaopeng Zhou, Yiqing Tao, Chengzhen Liang, Yujie Zhang, Hao Li, Qixin Chen

**Affiliations:** Department of Orthopedic Surgery, 2nd Affiliated Hospital, School of Medicine, Zhejiang University, 88 Jiefang Road, 310009 Hangzhou, China; E-Mails: doctorzxp@zju.edu.cn (X.Z.); xellosspeach@hotmail.com (Y.T.); liangchengzhen@gmail.com (C.L.); zycx@zju.edu.cn (Y.Z.); spinelihao@gmail.com (H.L.)

**Keywords:** differentiation, mesenchymal stem cells, transforming growth factor-β, bone morphogenetic protein-3, nucleus pulposus-like cells

## Abstract

Human mesenchymal stem cells (MSCs) have the potential to differentiate into nucleus pulposus (NP)-like cells under specific stimulatory conditions. Thus far, the effects of bone morphogenetic protein 3 (BMP3) and the cocktail effects of BMP3 and transforming growth factor (TGF)-β on MSC proliferation and differentiation remain obscure. Therefore, this study was designed to clarify these unknowns. MSCs were cultured with various gradients of BMP3 and BMP3/TGF-β, and compared with cultures in basal and TGF-β media. Cell proliferation, glycosaminoglycan (GAG) content, gene expression, and signaling proteins were measured to assess the effects of BMP3 and BMP3/TGF-β on MSCs. Cell number and GAG content increased upon the addition of BMP3 in a dose-dependent manner. The expression of *COL2A1*, *ACAN*, *SOX9*, and *KRT19* increased following induction with BMP3 and TGF-β, in contrast to that of *COL1A1*, *ALP*, *OPN*, and *COMP*. Smad3 phosphorylation was upregulated by BMP3 and TGF-β, but BMP3 did not affect the phosphorylation of extracellular-signal regulated kinase (ERK) 1/2 or c-Jun N-terminal kinase (JNK). Our results reveal that BMP3 enhances MSC proliferation and differentiation into NP-like cells, as indicated by increased cell numbers and specific gene expressions, and may also cooperate with TGF-β induced positive effects. These actions are likely related to the activation of TGF-β signaling pathway.

## 1. Introduction

The intervertebral disc (IVD) is composed of a highly hydrophilic central nucleus pulposus (NP) surrounded by the annulus fibrosus (AF) and cartilaginous endplate [1]. Degeneration of IVD occurs predominantly in the NP, which is abundant in proteoglycan, aggrecan, and type II collagen. Studies have revealed two main cell types within the NP, small chondrocyte-like cells and large vacuolated cells derived from the notochord [2]. The disappearance of NP-like cells due to various reasons leads to a loss of extracellular matrix proteins, thereby influencing the structural integrity of the NP, and thus initiating the IVD degeneration [3].

Cell-based tissue engineering is being widely evaluated to repair the degenerating IVD. Human mesenchymal stem cells (MSCs) are adult tissue-derived progenitor cells with the ability to differentiate into various cell types [4]. Several reports have demonstrated that MSCs can be guided into the NP-like phenotype under specific culture conditions, including the presence of growth factors, three-dimensional scaffold, and hypoxia among others [5,6].

Transforming growth factor (TGF)-β is a traditional growth factor with the ability to enhance proteoglycan and type II collagen synthesis in NP cells [7,8], and it is capable of inducing MSCs into a NP-like phenotype [9,10]. However, the differentiation efficiency is insufficient for tissue-engineering, because TGF-β also has the ability to induce differentiation of MSCs into chondrocytes or osteocytes [11,12].

Bone morphogenetic protein3 (BMP3) is a member of the TGF-β superfamily [13] and is present in large quantities within bone [14]. BMP3 plays an important role in fracture healing and mechanical loading of the skeleton [15]. Some studies revealed that BMP3 is anti-osteogenic and an antagonist of osteogenic BMPs [13,16]. Recently, one relevant study demonstrated that BMP3 stimulates MSC proliferation via the TGF-β/activin signaling pathway [17]. However, the effect of BMP3 on MSC differentiation has not been elucidated. TGF-β promotes MSC proliferation and differentiation mainly through the TGF-β, extracellular-signal regulated kinase (ERK) and c-Jun N-terminal kinase (JNK) signaling pathways among others [6,18,19,20]. BMP3 exerts its effect on cells by activating the TGF-β responsive receptor rather than the BMP-responsive receptor [16], in a manner similar to that of TGF-β. Presently, the signaling pathway affected by BMP3 and the co-effect of TGF-β and BMP3 on MSCs remain unclear, which should be addressed further.

We designed this study in order to find more efficacious ways to guide MSCs differentiate into NP-like cells in vitro. BMP3 and TGF-β provide us new ideas. Now we sought to determine the effects of BMP3 and cocktail effects of BMP3/TGF-β on proliferation and differentiation of MSCs. With more NP-like cells we will have more opportunity to restore the degenerative IVD by cells transplantation. Experiments were carried out in triplicate to ensure the reliability of results.

## 2. Results

### 2.1. Cell Proliferation

**Figure 1:**
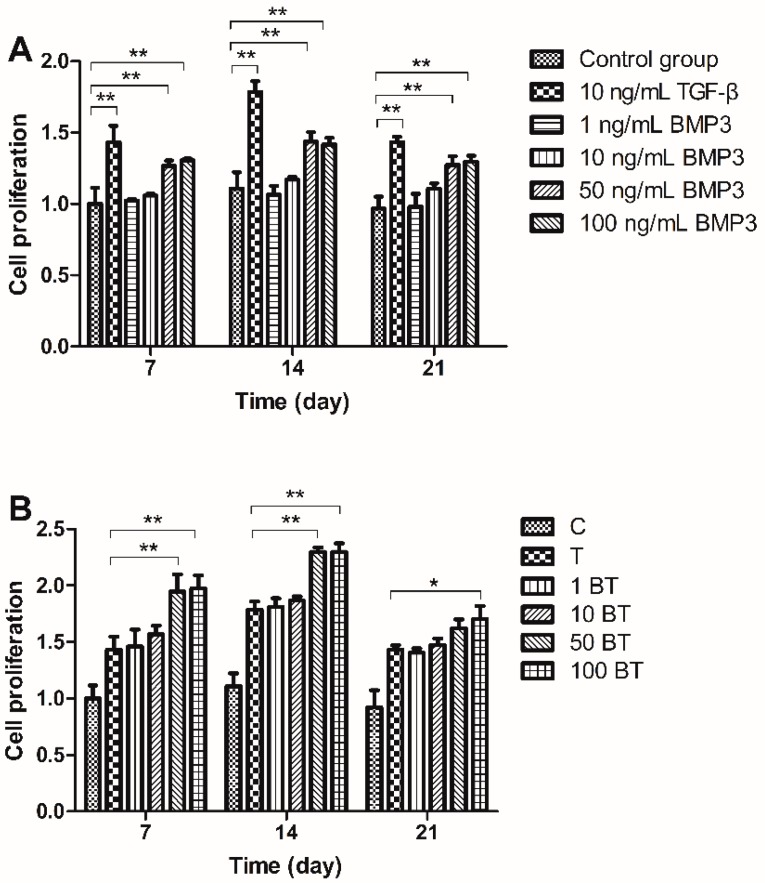
Effect of TGF-β, BMP3 and different gradients of BMP3/TGF-β on cell proliferation on days 7, 14, and 21. (**A**) Effect of 10 ng/mL TGF-β and different gradients of BMP3 on mesenchymal stem cells (MSCs) proliferation and (**B**) Effect of different gradients of BMP3/TGF-β on MSCs proliferation. C = control; T = 10 ng/mL TGF-β; 1 BT = 1 ng/mL BMP3/10 ng/mL TGF-β; 10 BT = 10 ng/mL BMP3/10 ng/mL TGF-β; 50 BT = 50 ng/mL BMP3/10 ng/mL TGF-β; 100 BT = 100 ng/mL BMP3/10 ng/mL TGF-β. Data represent mean ± SD; * *p* < 0.05, ** *p* < 0.01.

We measured the amount of cellular proliferation with Cell Counting Kit-8 (CCK-8) for its high sensitivity and low cytotoxicity. Cell proliferation was upregulated with the stimulation of TGF-β compared with control group (C). BMP3 alone (B) upregulated cell proliferation especially at the concentration of 50 and 100 ng/mL (Figure 1A). MSCs exposed to 1 ng/mL BMP3 and 10 ng/mL TGF-β (1 BT) or to 10 ng/mL BMP3 and 10 ng/mL TGF-β (10 BT) showed a similar proliferation level to those treated with TGF-β alone (T), indicating that the concentrations of BMP3 used were not sufficient for increasing cell proliferation. Groups treated with 50 ng/mL BMP3/10 ng/mL TGF-β 50 BT) and 100 ng/mL BMP3/10 ng/mL TGF-β (100 BT) exhibited greater cell proliferation than that of group T; however, there was no significant difference between groups 50 BT and 100 BT. The proliferation trend of day 7, 14 and 21 were similar. Cell proliferation increased from day 7 to 14 and decreased from day 14 to 21; therefore, MSCs may exhibit the highest cell number on day 14 (Figure 1B).

### 2.2. Glycosaminoglycan (GAG) Content

**Figure 2:**
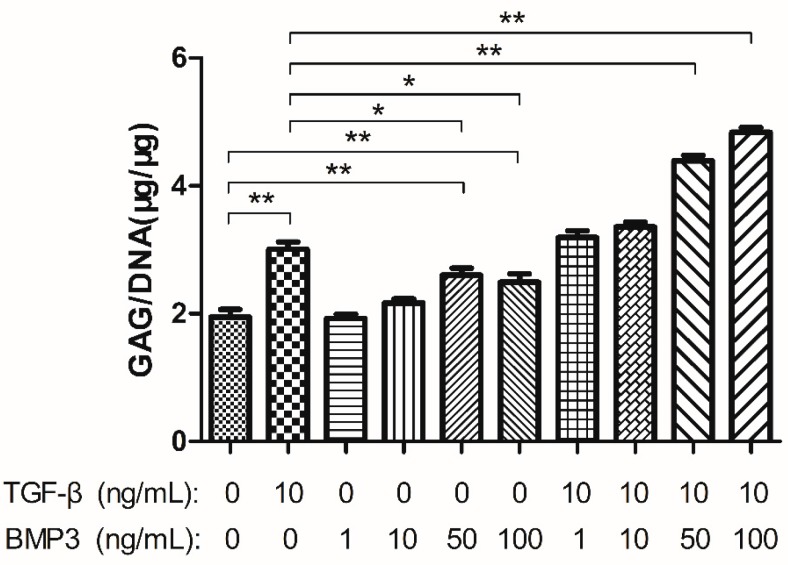
Glycosaminoglycan (GAG) synthesis normalized to DNA content of each group after cultured for 14 days. Data represent mean ± SD; * *p* < 0.05; ** *p* < 0.01.

GAG content was measured in order to evaluate the matrix synthesis of cells. As the CCK8 results demonstrated that the cell number on day 14 was the largest, GAG production was measured over the first 14 days. The GAG to DNA ratio was used for comparisons. There was no significant difference between group C and groups treated with 1 ng/mL BMP3 (1 B) and 10 ng/mL BMP3 (10 B), though when the concentration increased to 50 ng/mL (50 B) and 100 ng/mL (100 B), GAG production upregulated significantly. Group T demonstrated a higher GAG:DNA ratio compared with group C, groups 50 B and 100 B, but was similar to those of groups 1 BT and 10 BT (*p* > 0.05). As the BMP3 concentration increased to 50 ng/mL and cooperated with TGF-β, the GAG:DNA content increased synchronously (more than 2-fold). Group 100 BT expressed higher GAG:DNA content; however, the range between groups 50 BT and 100 BT was dramatically less than that between group 10 BT and 50 BT (Figure 2).

### 2.3. Gene Expression Analysis

**Figure 3:**
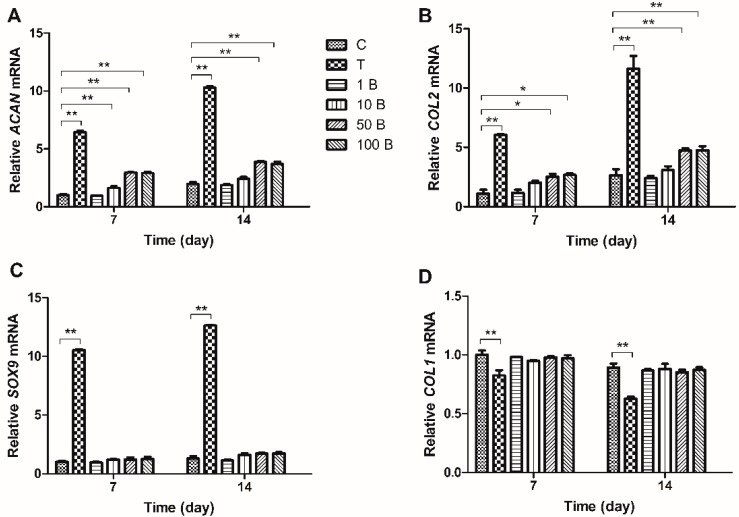
Relative gene expressions of MSCs on days 7 and 14. 10 ng/mL TGF-β, 1, 10, 50 and 100 ng/mL BMP3 were designed to clarify the effect of BMP3 alone on the expression of functional gene markers. Expression of (**A**) *ACAN*; (**B**) *COL2A1*; (**C**) *SOX9* and (**D**) *COL1A1* was normalized to *18S* and to the day 7 control group. Data represent mean ± SD; * *p* < 0.05, ** *p* < 0.01.

**Figure 4:**
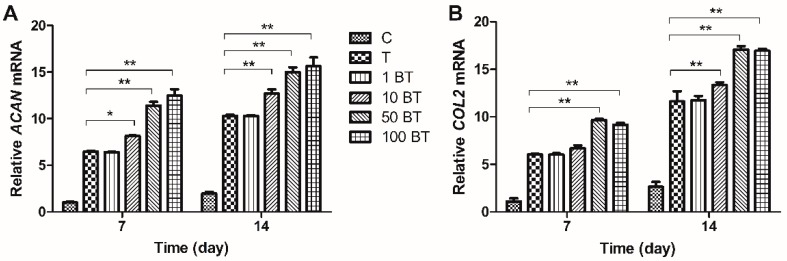

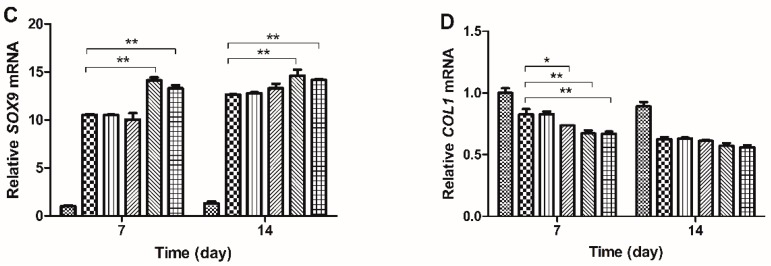
Relative gene expressions of MSCs on days 7 and 14. Groups 1 BT, 10 BT, 50 BT and 100 BT were designed to clarify the effect of BMP3/TGF-β on the expression of functional gene markers. Expression of (**A**) *ACAN*; (**B**) *COL2A1*; (**C**) *SOX9* and (**D**) *COL1A1* was normalized to *18S* and to the day 7 control group. C = control; T = 10 ng/mL TGF-β; 1 BT = 1 ng/mL BMP3/10 ng/mL TGF-β; 10 BT = 10 ng/mL BMP3/10 ng/mL TGF-β; 50 BT = 50 ng/mL BMP3/10 ng/mL TGF-β; 100 BT = 100 ng/mL BMP3/10 ng/mL TGF-β. Data represent mean ± SD; * *p* < 0.05, ** *p* < 0.01.

Real-time PCR was employed to evaluate changes in gene expression. mRNA expression of *ACAN*, *COL2A1* and *SOX9* was upregulated in MSCs treated with TGF-β on both days 7 and 14. BMP3 alone increased the expression of *ACAN* and *COL2A1*, especially on day 14, although not to the same degree as did TGF-β. The up-regulation was most significant on the concentration of 50 ng/mL (*ACAN* 2.9-fold and *COL2A1* 2.5-fold) and 100 ng/mL (*ACAN* 2.9-fold and *COL2A1* 2.7-fold) and the differences between group 50 B and group 100 B were not significant (*p* > 0.05). The expression of *SOX9* could not be influenced by BMP3 alone (Figure 3A–C). TGF-β together with BMP3 increased the expression of *ACAN*, *COL2A1*, and *SOX9*, in a BMP3 concentration dependent manner. The expression of these markers was upregulated, especially at a BMP3 concentration of 50 ng/mL (more than 10-fold, *p* < 0.01). Groups 50 BT and 100 BT had similar expression levels of *ACAN*, *COL2A1* and *SOX9* on both days 7 and 14 (*p* > 0.05). Compared with day 7, the mRNA levels of *ACAN*, *COL2A1*, and *SOX9* were higher on day 14 in each group (Figure 4A–C). *COL1A1* showed nearly opposite results relative to *ACAN*, *COL2A1*, and *SOX9*. Groups with the addition of TGF-β expressed lower *COL1A1* compared with group C on both days 7 (0.82-fold) and 14 (0.7-fold). BMP3 alone did not affect the expression of *COL1A1* (*p* > 0.05), but combined with TGF-β, *COL1A1* decreased gradually with increasing BMP3 concentrations compared with group T on day 7 (from 0.89-fold decreased to 0.81-fold). However, *COL1A1* expression in the groups treated with BMP3/TGF-β did not show any significant difference (*p* > 0.05) on day 14 (Figure 3D and Figure 4D).

**Figure 5:**
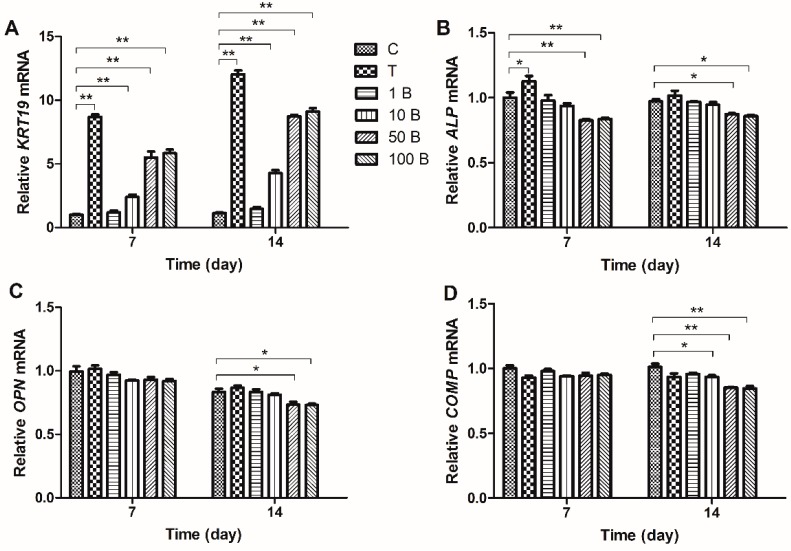
Expressions of differentiation related gene markers of MSCs treated with 10 ng/mL TGF-β and different gradients of BMP3 on days 7 and 14. Expression of novel NP markers (**A**) *KRT19*, and characteristic markers of osteocytes (**B**) *ALP*; (**C**) *OPN* and (**D**) *COMP* were normalized to *18S* and to the day 7 control group. Data represent mean ± SD; * *p* < 0.05, ** *p* < 0.01.

**Figure 6:**
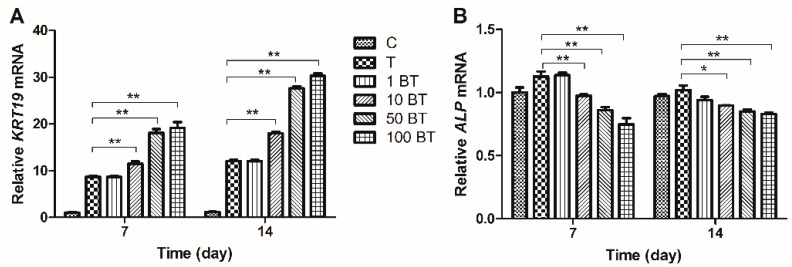

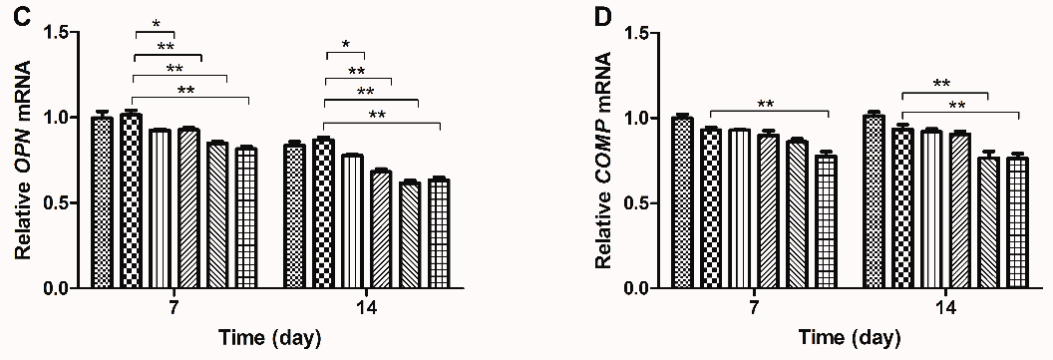
Expressions of differentiation related gene markers of MSCs treated with different gradients of BMP3/TGF-β on days 7 and 14. Expression of novel NP markers (**A**) *KRT19*, and characteristic markers of osteocytes (**B**) *ALP*; (**C**) *OPN* and (**D**) *COMP* were normalized to *18S* and to the day 7 control group. C = control; T = 10 ng/mL TGF-β; 1 BT = 1 ng/mL BMP3/10 ng/mL TGF-β; 10 BT = 10 ng/mL BMP3/10 ng/mL TGF-β; 50 BT = 50 ng/mL BMP3/10 ng/mL TGF-β, 100 BT = 100 ng/mL BMP3/10 ng/mL TGF-β. Data represent mean ± SD; * *p* < 0.05, ** *p* < 0.01.

*KRT19* is a characteristic gene of NP-like cells, and *OPN*, *COMP*, and *ALP* are characteristic genes of osteocytes, which can be used to indicate MSC differentiation. Our results showed that both TGF-β and BMP3 increased *KRT19* expression, and TGF-β exerting a stronger effect than BMP3. The minimum effective concentration of BMP3 was 10 ng/mL, group 50 B showed higher expression of *KRT19* than group 10 B, and the facilitating effects did not show any significant difference between group 50 B and group 100 B. Groups 50 BT and 100 BT had significantly higher expression of *KRT19* than those of the other groups on both days 7 and 14 (*p* < 0.01). There was no obvious difference between groups 50 BT and 100 BT on day 7 (*p* > 0.05), but a significant difference was detected on day 14 (*p* < 0.01) (Figure 5A and Figure 6A). TGF-β exhibited a trend of increasing *ALP* expression (1.2-fold compared with group C), which was downregulated by BMP3 at the concentration of 50 and 100 ng/mL. BMP3 also had the ability to reverse the TGF-β induced increase, especially on day 7 (Figure 5B and Figure 6B). TGF-β did not affect the expression of *OPN* at any time point. However, BMP3 downregulated the *OPN* mRNA levels alone (0.88-fold when the concentration was 50 ng/mL) and in the presence of TGF-β (0.74-fold when the concentration of BMP3 was 50 ng/mL), especially on day 14 (Figure 5C and Figure 6C). BMP3 inhibited the expression of *COMP*, and this inhibitory effect was significant on day 14 (0.84-fold when the concentration was 50 ng/mL). With higher concentrations of BMP3 (<100 ng/mL), the inhibition of *COMP* expression was more significant, while BMP3 at concentrations of 50 and 100 ng/mL exhibited a similar effect (*p* > 0.05), which were similar at the presence of TGF-β (Figure 5D and Figure 6D).

### 2.4. Western Blot Results

**Figure 7:**
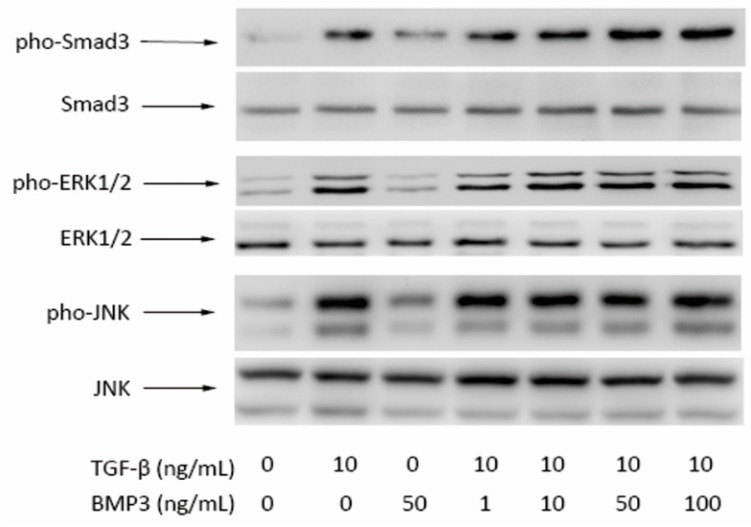
Western Blot of pho-Smad3/Smad3, (pho-ERK1/2)/(ERK1/2) and pho-JNK/JNK in protein extracts from each group of MSCs in beads after 4 h of culture.

Western blotting was performed to analyze the expression of Smad3, pho-Smad3, ERK1/2, pho-ERK1/2, JNK, and pho-JNK proteins, which are involved in TGF-β, ERK, and JNK signaling pathways. Each growth factor-supplemented construct showed increased expression of pho-Smad3 compared with group C, and the effect of TGF-β was stronger than that of BMP3. With increasing BMP3 concentrations, the expression of pho-Smad3 increased relatively. There was no significant difference in the expression of pho-ERK1/2 between groups C and 50 B. Group 1 BT, 10 BT, 50 BT, and 100 BT showed similar levels of protein expression with group T. The tendency of pho-JNK was similar to that of pho-ERK1/2. Levels of Smad3, ERK1/2 and JNK did not differ significantly among groups (Figure 7).

## 3. Discussion

Pre-differentiation of MSCs may provide a potential method for disc regeneration. Growth factors are widely used to promote MSC differentiation. BMP3 is abundant in bone; however, it has not been well-investigated, and the effects of BMP3 on MSC proliferation and differentiation remain unclear. In order to find a better method to guide MSC differentiation into NP-like cells, we examined the biological effect of BMP3 and the cocktail effects of BMP3 and TGF-β on MSCs.

Our study was carried out at an environment of 37 °C with 5% CO_2_ and 2% O_2_, since hypoxia has shown positive effects on directing the differentiation of MSCs towards the nucleus pulposus phenotype [21]. The positive effect of TGF-β on proliferation and matrix synthesis of MSCs have been demonstrated by us. BMP3 also plays a positive role in MSC proliferation, albeit with a weaker effect that of than TGF-β. This stimulatory effect is reinforced in the presence of both BMP3 and TGF-β. However, the additive effect is not limitless. A 5:1 (BMP3:TGF-β) concentration ratio exerts a similar effect as that of a 10:1 ratio on cellular proliferation and GAG synthesis. Samples from day 14 exhibited a higher CCK-8 index than those from days 7 and 21, suggesting that 14 days of treatment is the most beneficial for MSC proliferation.

Classic gene markers (*ACAN*, *COL2A1*, *COL1A1*, and *SOX9*), novel NP marker (*KRT19*) and characteristic osteocyte markers (*OPN*, *COMP*, and *ALP*) were measured to explore MSC differentiation. *KRT19* expression has the potential to characterize human NP cells and to distinguish them from chondrocytes and osteocytes [22]. *OPN*, *COMP*, and *ALP* are markers of MSC osteogenic differentiation. We discovered that the expression of *ACAN*, *COL2A1*, and *SOX9* increased following induction with BMP3 (except *SOX9*) and TGF-β. In contrast to that of *COL1A1*, these effects were also reinforced by co-treatment with BMP3 and TGF-β. Following treatment with TGF-β and BMP3, the expression of *KRT19* increased, whereas that of *ALP*, *COMP*, and *OPN* decreased, suggesting that BMP3 is able to induce MSC differentiation into NP-like cells instead of osteocytes. *ALP* is an early marker of osteogenic differentiation, whereas *OPN* and *COMP* are late markers of osteogenic differentiation [23]. TGF-β had no significant effect on the expression of *OPN* or *COMP*, but increased *ALP* expression on day 7, indicating that TGF-β promotes osteogenic differentiation in a certain degree. However, this effect could be reversed by certain concentrations of BMP3, such as 50 ng/mL. We conclude that BMP3 promotes TGF-β induced differentiation into NP-like cells and inhibits TGF-β induced differentiation into osteocytes. The cooperation between TGF-β and BMP3 stimulates more MSCs to differentiate into NP-like cells and increases the possibility of MSC-based IVD regeneration.

Our results indicated that BMP3 alone can enhance MSC’s proliferation and differentiate into NP-like cells. In order to find the most efficacious concentration, we set up a series of gradients. BMP3 increased cell proliferation, GAG production and the expression of *ACAN*, *COL2A1*, *SOX9* and *KRT19* from the concentration of 1 to 50 ng/mL. However, when we went on to increase the concentration of BMP3 to 100 ng/mL, no significant difference was observed. We conclude that BMP3 is saturated at the concentration of nearly 50 ng/mL, and continuing to increase the concentration cannot promote its effects.

To determine the most suitable ratio of TGF-β and BMP3 for differentiation, a series of gradients were examined. We used 10 ng/mL of TGF-β for comparison because this concentration of TGF-β has already been demonstrated to be suitable [24]. Treatment with 1 ng/mL BMP3 plus 10 ng/mL TGF-β had a similar effect as that of 10 ng/mL TGF-β alone, BMP3 did not appear to have any effect. When the BMP3 concentration was increased to 10 and 50 ng/mL, the cocktail effect of TGF-β and BMP3 increased relatively. We continued to increase the concentration of BMP3 to 100 ng/mL; however, gene expression analysis did not reveal any significant differences compared with 50 ng/mL BMP3. Studies have reported that BMP3 actives the TGF-β-responsive receptor rather than the BMP-responsive receptor [16]; therefore, we believe that the cocktail effect did not increase relatively as the concentration of BMP3 was increased from 50 to 100 ng/mL, because the combination of growth factors and TGF-β receptors caused saturation. BMP3 was superfluous at a concentration of 100 ng/mL.

We further investigated the molecular mechanism of several relevant signaling pathways. Smad3 is an important factor in the TGF-β signaling pathway and is regarded as a crossover point between the TGF-β signaling pathway and others, such as wnt signaling pathway [25]. Western blot analysis revealed that Smad3 phosphorylation was increased by BMP3, whereas total Smad3 levels were essentially unchanged. Moreover, the phosphorylation levels of Smad3 were upgraded relatively with increasing concentrations of BMP3. Therefore, BMP3 accelerated Smad3 phosphorylation not only in normal cells, but also in TGF-β induced MSCs, in a dose-dependent manner. The levels of phosphorylated ERK1/2 and JNK were dramatically increased following treatment with TGF-β, but did not change by the addition of BMP3. In addition, the presence of BMP3 did not affect TGF-β induced phosphorylation of ERK1/2 or JNK. Cross-talk between TGF-β/Smad and integrin-β1/FAK/ERK signaling in the downstream components has already been reported [26]. As these downstream components were not evaluated in our study, we can only assume that BMP3 does not influence the upstream components of the ERK and JNK signaling pathways. BMP3 and TGF-β interact with the same receptors but just partly exert the same effects.

Some studies support the facts that Smad3-dependent TGF-β signal pathway is an important event in MSC proliferation and differentiation [27,28]. Therefore it is possible that activation of the TGF-β signaling pathway promotes MSC proliferation and differentiation into NP-like cells. In our study, we did not obtain any direct information regarding the molecular mechanism of osteogenic differentiation suppression. However, we can exclude the possibility of a role of ERK1/2 or JNK, as BMP3 did not alter their phosphorylation. 

## 4. Experimental Section

### 4.1. Cell Culture

**Figure 8:**
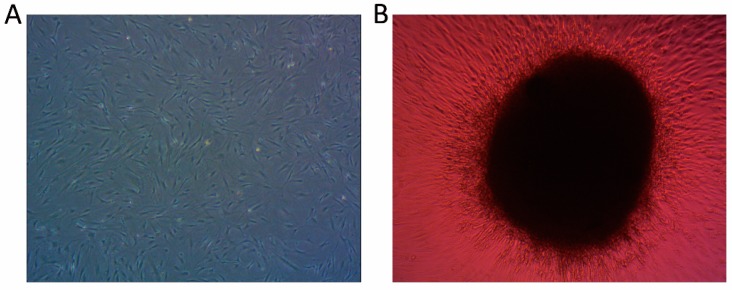
(**A**) Human MSCs were expanded in monolayer culture (40×) and (**B**) Cell pellet before treating with growth factors (40×).

Human adult MSCs of Section 2 were purchased from Cyagen Biosciences (Guangzhou, China), human adult MSC complete culture medium (human adult MSC basal culture medium supplemented with 10% fetal bovine serum (FBS), 1% penicillin-streptomycin solution and 1% glutamine) bought from Cyagen Biosciences (Guangzhou, China) was used for MSC cultivation. All cells were maintained in a humidified incubator at 37 °C and 5% CO_2_ prior to treatment, media were replaced twice per week (Figure 8A). Cell of passages 5–7 were used for subsequent experiments.

### 4.2. Pellets and Cell Differentiation

Pellet system was utilized for MSC culture, as 3-dimensional pellet culture systems are able to increase matrix synthesis and gene expressions. Cell suspensions containing approximately 2.5 × 10^5^ cells were centrifuged at 500× *g* for 5 min in 15 mL polypropylene conical tubes contain 1 mL complete culture medium. Pellets were then incubated in a humidified incubator at 37 °C and 5% CO_2_ for 24 h until microspheres formed [29]. The pellets were then transferred to 24-well plates with one pellet in each well (Figure 8B), and Dulbecco’s modified Eagle’s medium/high glucose supplemented with 1% FBS, 6.25 µg/mL insulin, 50 nM ascorbate-2-phospate, 1% penicillin-streptomycin and growth factors was added. The 10 separate groups used for analysis were designed as follows: (1) Group C: no additional growth factors; (2) Group T: addition of 10 ng/mL TGF-β; (3) Group 1 B: addition of 1 ng/mL BMP3; (4) Group 10 B: addition of 10 ng/mL BMP3; (5) Group 50 B: addition of 50 ng/mL BMP3; (6) Group 100 B: addition of 100 ng/mL BMP3; (7) Group 1 BT: addition of 1 ng/mL BMP3 and 10 ng/mL TGF-β; (8) Group 10 BT: addition of 10 ng/mL BMP3 and 10 ng/mL TGF-β; (9) Group 50 BT: addition of 50 ng/mL BMP3 and 10 ng/mL TGF-β; (10) Group 100 BT: addition of 100 ng/mL BMP3 and 10 ng/mL TGF-β. Each group was incubated at 37 °C with 5% CO_2_ and 2% O_2_ [21], and media were changed twice per week. Samples were collected at 7, 14, and 21 days.

### 4.3. Measurement of Cellular Proliferation

Day 7, 14, and 21 samples were collected to determine the amount of cellular proliferation. Pellets were transferred to a 96-well plate, and 100 µL prepackaged CCK-8 (Dojindo Molecular Technologies, Kumamoto, Japan) solution was used to react. After incubation at 37 °C with 5% CO_2_ for 2 h, 80 µL supernatant from each well were transferred to another 96-well plate. Absorbance values at 450 nm were measured by a spectrophotometer. Results are expressed as percentages of the control group on day 7.

### 4.4. Measurement of Glycosaminoglycan Synthesis

Day 14 samples were collected for the measurement of GAG synthesis. Dimethylmethylene blue (DMMB) and a standard curve were used to assess the amount of GAG released into the media. 20 µL media samples were mixed with 200 µL DMMB dye reagent in a 96-well plate and incubated in a culture apparatus at 37 °C with 5% CO_2_ for 30 min. Absorbance values at 525 nm were measured using a spectrophotometer. The DNA content of each pellet was measured using Hoechst 33258 and compared on a standard curve. Cell pellets were first treated with 125 µg/mL papain at 60 °C for 24 h, then centrifuged at 10,000× *g* for 15 min, and 100 µg suspensions were mixed with 100 µg Hoechst 33258 in a 96-well plate and incubated in a culture apparatus at 37 °C with 5% CO_2_ for 30 min. Absorbance was measured at excitation 365 nm/emission 460 nm wavelengths using a multimode reader. The amount of GAG was then normalized to the DNA content.

### 4.5. Real Time Quantitative-PCR

RNA was extracted from pellets (*n* = 3) of day 7 and 14 using TRIzol reagent after complete grinding. Total RNA was used to synthesize cDNA utilizing a Double-Strand cDNA Synthesis Kit (TAKARA, Dalian, China) according to the manufacturer’s instructions. cDNAs were diluted 1:4 with RNase-free water and stored at −20 °C until use. SYBR Green PCR assays (TAKARA) were used to perform real-time PCR, and three independent samples were set to ensure validity. *18S* rRNA was used as the housekeeping gene, and an additional eight target genes were detected (Table 1). Primers were synthesized by Sangon Biotech (Shanghai, China), and quantitative real-time PCR data were calculated by the 2 ^−ΔΔ*C*t^ method [30].

**Table 1:** Primers used in quantitative RT-PCR. (F = Forward, R = Reverse).

Gene	Primer Nucleotide Sequence (5′ to 3′)	Product Size (bp)
*18S*	F-ATCCTCAGTGAGTTCTCCCG	106
	R-CTTTGCCATCACTGCCATTA	
*ACAN*	F-AGAATCAAGTGGAGCCGTGT	115
	R-GGTAGTTGGGCAGTGAGACC	
*SOX9*	F-AGCGAACGCACATCAAGAC	129
	R-CTGTAGGCGATCTGTTGGGG	
*KRT19*	F-GATAGTGAGCGGCAGAATCA	178
	R-CCTCCAAAGGACAGCAGAAG	
*COL2A1*	F-CATCCCACCCTCTCACAGTT	151
	R-ACCAGTTAGTTTCCTGCCTCTG	
*COL1A1*	F-AGTCTGTCCTGCGTCCTCTG	183
	R-TGTTTGGGTCATTTCCACAT	
*ALP*	F-TTTATAAGGCGGCGGGGGT	142
	R-TTAACTGATGTTCCAATCCTGCG	
*COMP*	F-CAACCAGGGAAGGGAGATCG	130
	R-CGCATAGTCGTCATCCGTGA	
*OPN*	F-AGCAGCTTTACAACAAATACCCAG	100
	R-TTACTTGGAAGGGTCTGTGGG	

### 4.6. Western Blot Analysis

Cell pellets were starved in serum-free medium for 24 h, and treated with the indicated conditions/times thereafter. Samples were washed three times with ice-cold phosphate-buffered saline (PBS), and total proteins were extracted with RIPA buffer containing 1% PMSF. Protein concentrations were measured using a BCA Protein Quantification Kit (Pierce, New York, NY, USA). Proteins were electrophoresed by 10% sodium dodecyl sulfate polyacrylamide gel electrophoresis (SDS-PAGE) and then transferred onto polyvinylidene fluoride (PVDF) membranes (Millipore, Massachusetts, MA, USA). After blocking with 5% skim milk in Tris-buffered saline with 0.1% Tween-20 (TBST) at room temperature for 1 h, membranes were hybridized overnight at 4 °C in TBST with the appropriate primary antibody: anti-JNK antibody (1:1000; #9258, Cell Signaling Technology, Danvers, MA, USA), anti-p-JNK antibody (1:1000; #4668, Cell Signaling Technology), anti-Smad3 antibody (1:1000; #9523, Cell Signaling Technology), anti-p-Smad3 antibody (1:1000; #9520, Cell Signaling Technology), anti-ERK1/2 antibody (1:8000; SC-135900, Santa Cruz Biotechnology, Dallas, Texas, USA), anti-p-ERK1/2 antibody (1:8000; SC-16981-R, Santa Cruz Biotechnology), or anti-β-actin antibody (1:2000; Pierce, New York, NY, USA). Membranes were then incubated with horseradish peroxidase (HRP)-labeled secondary IgG (1:5000; anti-mouse or anti-rabbit, Pierce) for 1 h at room temperature. After washing with TBST three times, immunoreactivity was detected with enhanced chemiluminescence (ECL) substrate, and densitometry was performed using QuantityOne Software (Bio-Rad Laboratories Inc., Munich, Germany).

### 4.7. Statistical Analysis

All data are expressed as the mean ± standard deviation (SD). The SPSS software package (Version 16.0, SPSS Inc., Chicago, IL, USA) was used for statistical analysis. Statistical significance was determined using Student’s *t*-test and one-way analysis of variance (ANOVA). A value of *p* < 0.05 was considered to indicate statistical significance. Each experiment was repeated three times.

## 5. Conclusions

In the study described herein, the effects of both BMP3 alone and in combination with TGF-β on MSC proliferation and differentiation were analyzed, as well as the potential molecular mechanism. We found that BMP3 promotes MSC’s proliferation and demonstrates a synergistic effect with TGF-β. Our results suggest that BMP3 plays a positive role in the differentiation of MSCs into NP-like phenotype, as seen through the expression of the *KRT19* gene, as well as in anti-osteogenic differentiation. BMP3 may work together with TGF-β on MSC differentiation into NP-like cells, and the most efficacious concentration ratio is nearly 5:1 (BMP3:TGF-β). Results also indicate that BMP3 exerts its effect on MSCs through the TGF-β signaling pathway rather than the ERK1/2 and JNK. Additional studies should be pursued to further understand the influence of BMP3 on MSCs and discover more effective and practical therapies for the treatment of IVD degeneration.
